# Immunometabolic reprogramming in multiple sclerosis: from pathogenic amplifier to therapeutic target in neuroinflammation and remyelination

**DOI:** 10.1007/s10787-026-02350-y

**Published:** 2026-07-28

**Authors:** Ghada A. Badawi, Rehab M. El-Sayed, Mohamed N. Fawzy

**Affiliations:** Department of Pharmacology and Toxicology, Faculty of Pharmacy, Sinai University-Arish Branch, Arish, 45511 Egypt

**Keywords:** Multiple Sclerosis, Immunometabolism, Neuroinflammation, Drug repurposing, mTOR signaling, AMPK

## Abstract

Multiple sclerosis (MS) has conventionally been considered a prototypical autoimmune disorder. While modern immunotherapies effectively control relapsing conditions, they fail to avert progressive neuroinflammation, neurodegeneration, and remyelination failure. This review offers an evidence-based recontextualization. The pathogenesis of multiple sclerosis is multifactorial, resulting from the interaction of genetic predisposition (especially HLA alleles), environmental factors (particularly Epstein-Barr virus infection), B-cell-mediated autoimmunity, and localized CNS inflammation. In this intricate environment, cellular metabolic dysregulation is a notable factor and potentially adjustable enhancer of disease progression, functioning within the extensive multifactorial pathogenic context, where the bioenergetic programming of immune and neural cells determines inflammatory or protective responses. Proinflammatory Th17 cells and M1 microglia depend on aerobic glycolysis and glutaminolysis regulated by mTOR and HIF-1α, whereas regulatory T cells, M2 microglia, and neurons require fatty acid oxidation and oxidative phosphorylation via AMPK. In multiple sclerosis, glycolysis disrupts metabolic equilibrium, sustaining chronic neuroinflammation and obstructing repair processes. Established multiple sclerosis therapies, dimethyl fumarate and teriflunomide, exhibit direct, previously unrecognized metabolic effects, validating this pathway as therapeutically viable. Emerging strategies intentionally target these vulnerabilities: glutaminase inhibitors to counteract pathogenic Th17 cells, AMPK activators such as metformin to enhance remyelination, mTOR inhibitors to restore immune tolerance, and NAD + precursors to rejuvenate mitochondrial function. Transitioning from broad immunosuppression to specific metabolic reprogramming offers remarkable opportunities for tackling chronic neuroinflammation and correcting remyelination deficits in progressive multiple sclerosis. Immuno-metabolic pharmacology is a promising field; however, its clinical application necessitates stringent validation via meticulously designed trials and dependable biomarkers.

**Supplementary Information:**

The online version contains supplementary material available at https://doi.org/10.1007/s10787-026-02350-y.

## Introduction

Multiple sclerosis (MS) is a chronic, inflammatory, demyelinating, and neurodegenerative disorder of the central nervous system (CNS) and a leading cause of non-traumatic neurological disability in young adults (Abulaban et al. 2025; Al-Dhahi et al. 2025). For decades, our understanding of MS pathogenesis has been firmly rooted in an immunological paradigm. The prevailing model posits a subtle relationship between genetic susceptibility, particularly within the major histocompatibility complex (MHC) region, and environmental triggers, most notably Epstein-Barr virus infection, which together conspire to break immune tolerance (Boutitah-Benyaich et al. 2025). This breach unleashes a cascade of autoreactive immune cells, chief among them myelin-specific CD4 + T helper (Th) 1 and Th17 cells, along with CD8 + T cells and pathogenic B cells, that traverse the blood–brain barrier (BBB) to launch a sustained attack on the myelin sheath. The resulting neuroinflammation, characterized by focal white matter lesions, axonal transection, and gliosis, manifests clinically as the relapsing–remitting attacks that define the most common form of the disease (Boutitah-Benyaich et al. 2025; Zhu et al. 2025).

However, as the field has advanced, it has become increasingly clear that MS pathogenesis extends well beyond simple immune dysregulation. The disease process is fundamentally multifactorial, incorporating chronic active lesion formation, mitochondrial injury, iron-related oxidative toxicity, and compartmentalized inflammation within the CNS, each contributing to the relentless axonal degeneration and remyelination failure that drive progressive disability (Dal-Bianco et al. 2024).

This immunological framework has yielded substantial clinical advances from a therapeutic standpoint. Over the past three decades, the treatment landscape for relapsing–remitting MS (RRMS) has been transformed (Boutitah-Benyaich et al. 2025; Sabatino et al. 2025). The armamentarium of disease-modifying therapies (DMTs) has expanded from the first-line injectables (interferon-beta and glatiramer acetate) to a diverse array of highly effective agents. These include drugs that keep lymphocytes in lymph nodes (sphingosine-1-phosphate receptor modulators), stop immune cells from moving across the BBB (natalizumab), kill B cells that are already in the blood (anti-CD20 monoclonal antibodies), or cause apoptosis in lymphocytes that are growing (cladribine) (Sabatino et al. 2025; Rae-Grant et al. 2018). There is no doubt that these immunomodulatory and immunosuppressive strategies have worked. They have greatly lowered relapse rates, slowed the progression of disability, and made life better for many people with MS. The evolution of these therapies represents a major achievement of modern pharmacology and translational immunology (Ridley et al. 2025).

However, despite these remarkable advances, significant and sobering challenges remain. A substantial proportion of patients continue to experience breakthrough disease activity or fail to respond adequately to therapy. The pharmacological success achieved in RRMS has not been effectively translated to the progressive forms of the disease. Once patients transition to secondary progressive MS (SPMS) or present with primary progressive MS (PPMS), the focal inflammatory attacks diminish, and the clinical picture becomes dominated by a diffuse, ‘‘smoldering’’ neuroinflammation, compartmentalized behind a closed or repaired BBB, accompanied by relentless axonal loss and failure of remyelination (Sabatino et al. 2025; Brieva et al. 2025).

The core premise of this review is that the limitations of current MS pharmacotherapy stem, in part, from an incomplete understanding of the cellular state driving pathogenesis. We have focused intensely on the identity and trafficking of pathogenic immune cells, but we have largely overlooked the fundamental biology that fuels their pathogenic functions. The question is no longer just which cells are pathogenic but how they are bioenergetically programmed to sustain their destructive phenotype (Boutitah-Benyaich et al. 2025). This brings us to the burgeoning field of immunometabolism, which has revolutionized our understanding of immune cell fate and function. It is now well-established that immune cells do not merely utilize metabolism as a housekeeping function to generate ATP (Wang et al. 2025a).

Instead, specific metabolic pathways are dynamically rewired to support different cellular activities. A resting, naive T cell primarily relies on oxidative phosphorylation (OXPHOS) in the mitochondria, a highly efficient means of generating energy. Upon activation, a pro-inflammatory effector T cell (such as a Th17 cell) undergoes a profound metabolic switch, akin to the Warburg effect observed in cancer cells. This process increases aerobic glycolysis and glutaminolysis, even in the presence of abundant oxygen (Kates and Saibil 2024; Shi et al. 2025; Hong et al. 2022). This shift is not primarily for energy efficiency but to rapidly generate the biosynthetic precursors, nucleotides, amino acids, and lipids necessary for clonal expansion and effector molecule production. In stark contrast, immunosuppressive regulatory T cells (Tregs) and long-lived memory T cells maintain a preference for fatty acid oxidation (FAO) and OXPHOS, a metabolic profile that supports their longevity and suppressive functions (Shi et al. 2025; Michalek et al. 2011; Lu et al. 2025).

This metabolic programming is not a passive consequence but is tightly controlled by master regulators such as the mammalian target of rapamycin (mTOR), AMP-activated protein kinase (AMPK), and hypoxia-inducible factor 1 alpha (HIF-1α) (Abd El-Fattah et al. 2022; Zhu et al. 2024). We are now beginning to appreciate that this intricate immuno-metabolic axis is profoundly dysregulated in MS. The inflammatory milieu within the CNS and peripheral immune compartments actively drives and sustains the glycolytic, pro-inflammatory phenotype of autoreactive T cells and B cells (Shokr 2025; Sun et al. 2025a).

Simultaneously, this same environment can metabolically reprogram and destabilize Tregs, compromising their suppressive capacity. Furthermore, the story extends beyond infiltrating lymphocytes. In progressive MS, resident microglia and infiltrating macrophages appear to remain in a chronically activated, inflammatory state (Yamasaki 2025). These metabolic derangements do not operate in isolation; they interact with, and amplify, the broader pathogenic processes of mitochondrial dysfunction, iron accumulation, and failed repair mechanisms that characterize MS pathology. In this conceptualization, metabolic dysregulation acts as a central amplifier and modifiable hub within the complex network of MS pathogenesis, rather than the solitary primary cause.

This reframing suggests a new approach to pharmacological intervention. It suggests that the next generation of MS therapies should move beyond simply blocking cell movement or depleting cell populations. Instead, we can envision a strategy of immuno-metabolic reprogramming. The goal is to pharmacologically "rewire" the metabolic circuitry of pathogenic cells, forcing them away from a destructive, glycolytic state and towards a tolerant, reparative state. This perspective also provides a novel lens through which to re-evaluate our current DMTs. For instance, the therapeutic effects of dimethyl fumarate are now understood to be intimately linked to the activation of the nuclear factor erythroid 2-related factor 2 (Nrf2) pathway, a master regulator of antioxidant and metabolic defense (Oliveira 2026; Zingkou et al. 2026).

Teriflunomide's mechanism is a quintessential example of metabolic targeting: it selectively inhibits dihydroorotate dehydrogenase (DHODH), a key enzyme in the de novo pyrimidine synthesis pathway, thereby starving rapidly proliferating lymphocytes of the nucleotides they need to expand (Wu et al. 2023). Even established drugs like metformin, an AMPK activator used for type 2 diabetes, are now being explored for their potential to enhance Treg stability and promote neuroprotection in MS (Dziedzic et al. 2020). The convergence of these observations is striking and points to a central, unifying theme: metabolism is not a bystander in MS pathogenesis and therapy; it is a key driver, amplified by genetic and environmental factors, and a prime pharmacological target. By framing the challenges of breakthrough disease, progressive MS, and remyelination failure as fundamentally metabolic problems, we argue that pharmacologically rewiring the immuno-metabolic axis represents the most promising path forward to complement and enhance existing immunomodulatory strategies.

### Literature search strategy

This narrative review consolidates the literature on immunometabolic pharmacology in MS through a focused search of PubMed/MEDLINE, Scopus, and Web of Science for publications from January 2000 to March 2026, employing combinations of keywords such as ‘‘multiple sclerosis,’’ ‘‘immunometabolism,’’ ‘‘metabolic reprogramming,’’ ‘‘glycolysis,’’ ‘‘OXPHOS,’’ ‘‘mTOR, AMPK, HIF-1α, Th17 cells, Tregs,’’ ‘‘microglia, neuroinflammation, remyelination,’’ and pertinent pharmacological agents (e.g., dimethyl fumarate, teriflunomide, BTK inhibitors, and metformin). Articles were included if they examined metabolic pathways in immune or neural cells pertinent to MS pathogenesis, the function of metabolic regulators in immune responses, the metabolic impacts of MS therapies, or innovative metabolic targeting strategies; priority was assigned to peer-reviewed original research, high-quality reviews, and clinical studies, while conference abstracts, unpublished data, and non-English articles were excluded. Preclinical studies were incorporated that offered mechanistically translatable insights. The chosen topics and references demonstrate expert evaluation of significant contributions. Although we sought extensive thematic coverage, this review is narrative rather than systematic, excluding formal quality assessment to allow for integrative synthesis across various research domains and to promote hypothesis generation for therapeutic application.

## The emerging field of immuno-metabolism: foundational concepts

### Glycolysis vs. oxidative phosphorylation (OXPHOS): the warburg effect of immunity

The most fundamental metabolic distinction in immunology lies between glycolysis and oxidative phosphorylation. OXPHOS occurs in the mitochondria and represents the most efficient method for generating ATP from glucose. This process entails the complete oxidation of pyruvate, derived from glucose, through the tricarboxylic acid (TCA) cycle, which then feeds electrons into the electron transport chain (ETC). OXPHOS serves as the preferred energy source for quiescent, long-lived, and anti-inflammatory cells, including naive T cells, regulatory T cells (Tregs), and memory T cells. It optimizes energy yield per glucose molecule, thereby supporting cellular survival and longevity (Wang et al. 2020; Lopez 2024).

In contrast, aerobic glycolysis, first described by Otto Warburg in cancer cells, is the hallmark of rapidly proliferating and pro-inflammatory cells. Upon activation through their T-cell receptor and co-stimulation, effector T cells (such as Th1 and Th17 cells) undergo a dramatic metabolic switch (Menk et al. 2018; Haften et al. 2026; Li et al. 2025a). They upregulate glucose transporters (like GLUT1) and glycolytic enzymes, channeling glucose to lactate even in the presence of abundant oxygen. This process, at first glance, seems wasteful; it generates only 2 ATP per glucose molecule compared to up to 36 from OXPHOS (Lee et al. 2025).

However, the advantage lies in speed and biosynthesis. Aerobic glycolysis is a much faster way to generate ATP, and, crucially, it shunts glycolytic intermediates into branching biosynthetic pathways. For example, glucose-6-phosphate can enter the pentose phosphate pathway to generate ribose for nucleotide synthesis, while other intermediates provide precursors for amino acid and lipid synthesis. This allows an activated T cell to rapidly duplicate its entire cellular contents, a prerequisite for clonal expansion (Menk et al. 2018; Klarquist et al. 2018).

### Glutaminolysis and fatty acid metabolism: fuelling the immune response

Glucose is not the only critical fuel. Glutaminolysis is the process by which the amino acid glutamine is broken down. Upon activation, immune cells, particularly T cells and macrophages, massively increase their uptake of glutamine. Glutamine is a versatile fuel; it can be converted into glutamate and then into alpha-ketoglutarate to replenish the TCA cycle (a process called anaplerosis), ensuring that the cycle can continue to provide biosynthetic intermediates even as they are siphoned off for growth. Glutamine also donates nitrogen and carbon for the synthesis of nucleotides, amino sugars, and other essential molecules. For pro-inflammatory Th17 cells, glutaminolysis is absolutely critical for their differentiation and pathogenicity (Choi et al. 2025; Feng et al. 2022).

Lipids also play a dual role. Fatty acid synthesis (FAS) is essential for proliferating cells to build new membranes. This process is upregulated in activated effector T cells and is linked to their pro-inflammatory functions. Conversely, fatty acid oxidation (FAO) is the process of breaking down fatty acids in the mitochondria to generate large amounts of ATP, as well as NADH and FADH_2_. This pathway is the metabolic signature of cells with a suppressive or long-lived phenotype. Tregs, for instance, rely heavily on FAO to fuel their suppressive functions, and memory T cells utilize FAO to persist for years, providing rapid protection upon re-encounter with antigen. The balance between FAS and FAO is thus a key determinant of inflammatory versus regulatory immune outcomes (Zhang et al. 2024; Kemp et al. 2024).

### mTOR: the central growth and metabolism sensor

The mechanistic target of rapamycin (mTOR) is a serine/threonine kinase that functions as a central hub, integrating signals from growth factors, nutrients (amino acids, glucose), and energy status. It exists in two complexes: mTORC1 and mTORC2. mTORC1 is the primary metabolic rheostat. When nutrients are abundant and activating signals are present, mTORC1 is activated and potently drives an anabolic, pro-inflammatory program (Dhaliwal et al. 2026; Ragupathi et al. 2024). It does so by upregulating the transcription factors HIF-1α and MYC, which in turn increase the expression of glycolytic enzymes and glutamine transporters.

mTORC1 activity is essential for the differentiation of pro-inflammatory Th1 (T helper 1) and Th17 (T helper 17) cells, which are types of immune cells that promote inflammation. In contrast, it actively suppresses the generation of Tregs and memory T cells, which are important for maintaining immune tolerance and memory. Pharmacologically inhibiting mTOR with drugs like rapamycin can skew the immune response towards tolerance by promoting Treg differentiation and FAO (Shi et al. 2025, 2011; Chapman and Chi 2014).

#### AMPK: the cellular energy guardian

If mTOR is the accelerator of anabolism, AMP-activated protein kinase (AMPK) is the brake. AMPK is a highly conserved sensor of cellular energy status. It is activated by an increase in the AMP/ATP ratio, which signals low energy. Once activated, AMPK acts to restore energy balance by switching off all anabolic (energy-consuming) pathways and switching on catabolic (energy-producing) pathways. It achieves the latter goal by inhibiting mTORC1 and directly promoting mitochondrial biogenesis, glucose uptake, and, most importantly, FAO. By driving FAO and OXPHOS, AMPK promotes an anti-inflammatory, tolerogenic state. Activating AMPK with drugs like metformin or AICAR has been shown to suppress inflammatory T-cell responses and enhance Treg function, making it an extremely attractive therapeutic target for autoimmune diseases like MS (Garcia and Shaw 2017; Langer et al. 2024; Torres Acosta et al. 2025).

#### HIF-1α: the hypoxia and inflammation link

HIF-1α is a transcription factor classically known for its role in adapting cells to low oxygen (hypoxia). However, it is also a master driver of glycolysis. Under normoxic conditions, HIF-1α is usually hydroxylated and degraded. In activated immune cells, even with normal oxygen, mTORC1 signaling can stabilize HIF-1α, a phenomenon known as ‘‘pseudo-hypoxia.’’ Once stabilized, HIF-1α translocates to the nucleus and turns on the expression of nearly every gene in the glycolytic pathway (Ye et al. 2025; Basheeruddin and Qausain 2024).

It is absolutely critical for the differentiation and function of pro-inflammatory Th17 cells. HIF-1α also promotes the degradation of FoxP3, the master transcription factor of Tregs, thereby simultaneously promoting inflammation and inhibiting regulation. An inflammatory lesion’s metabolic environment, which can be both hypoxic and rich in inflammatory signals, provides the ideal conditions for HIF-1 activation, locking cells into a pathogenic, glycolytic state (Park and Ciofani 2025; Gu et al. 2024).

**Fig. 1:**
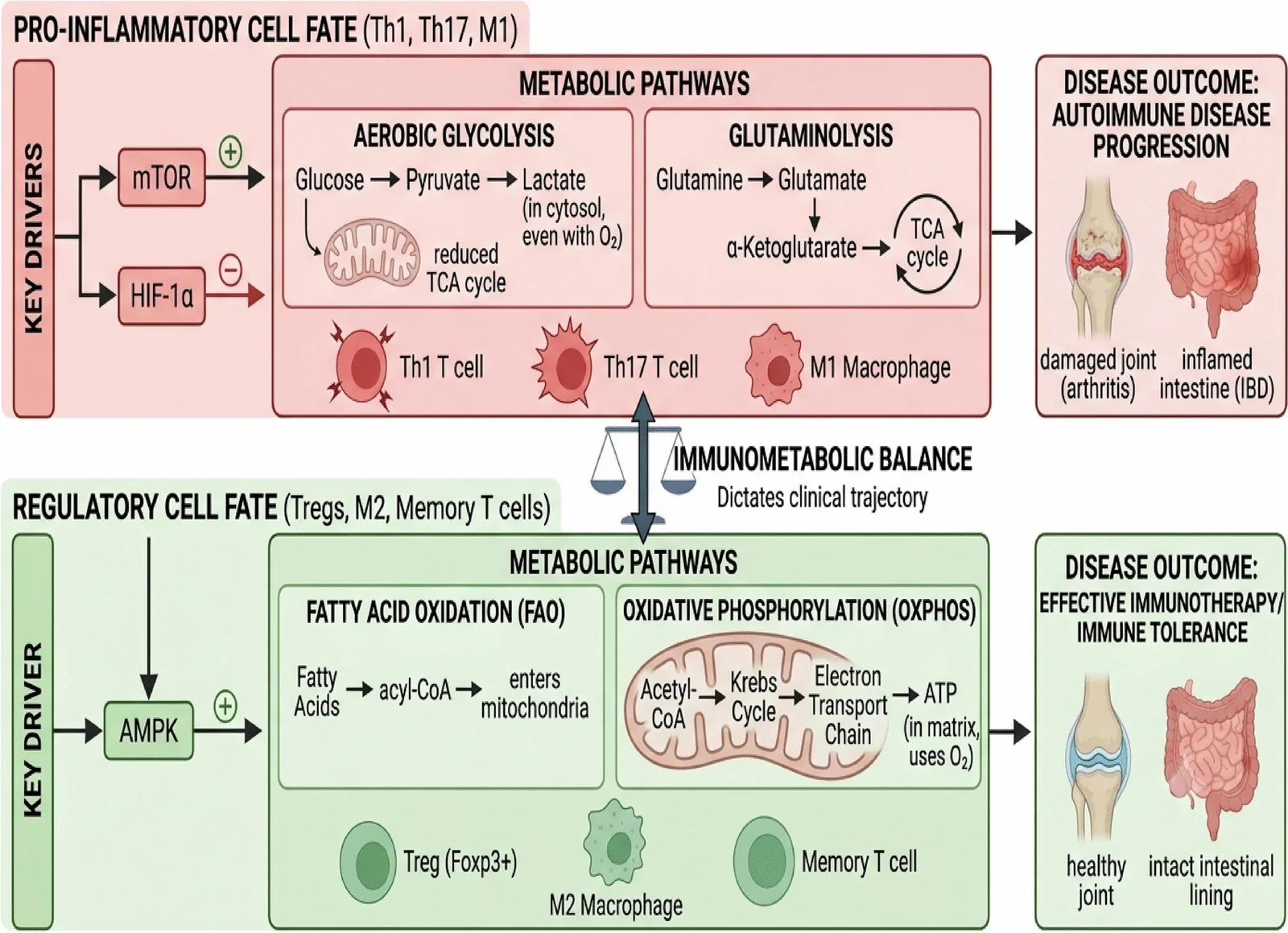
Opposing immunometabolic programs determine proinflammatory versus regulatory cell fate. Pro-inflammatory cells (Th1, Th17, and M1 macrophages) rely on aerobic glycolysis and glutaminolysis driven by mTOR and HIF-1α. Regulatory cells (Tregs, M2 macrophages, memory T cells) rely on fatty acid oxidation (FAO) and oxidative phosphorylation (OXPHOS) driven by AMPK. The balance between these opposing programs dictates autoimmune disease progression versus effective immunotherapy

Figure 1 summarizes the opposing metabolic programs of pro-inflammatory and regulatory immune cells and their master regulators. These foundational concepts provide the essential vocabulary and framework for understanding the immuno-metabolic axis in MS **[**Fig. 1**]**.

### The conceptual framework for MS

These foundational concepts provide the essential vocabulary and framework for understanding the immuno-metabolic axis in MS. In the next section, we will explore how the inflammatory milieu in the peripheral immune compartments and within the CNS of MS patients is not just a passive environment. It is an active metabolic niche that likely drives and sustains these very pathways, which are crucial for the functioning of immune cells and their interactions in MS. This niche promotes a glycolytic, HIF-1α-driven, mTOR-dependent program in autoreactive T cells and pro-inflammatory microglia, while simultaneously starving Tregs and oligodendrocyte precursor cells (OPCs) of the metabolic support they need to perform their suppressive and reparative functions (Wang et al. 2024).

Recognizing that this metabolic dysregulation does not occur in isolation is crucial. Rather, it interacts with and powerfully amplifies the established pathogenic drivers of MS: the pro-inflammatory cytokine milieu, oxidative stress, mitochondrial dysfunction, iron accumulation in chronic active lesions, and the compartmentalized inflammation that characterizes progressive disease (Peng et al. 2026). The metabolic state of immune and neural cells constitutes a pivotal interface where genetic predispositions (e.g., HLA variants affecting immune response efficacy) and environmental factors (e.g., EBV infection modifying metabolic requirements) intersect to influence disease progression. For instance, EBV-transformed B cells have altered metabolic profiles that may enhance their ability to present antigens and sustain pathogenic T-cell responses. Similarly, mitochondrial genetic variants may influence neuronal vulnerability to inflammatory metabolic stress (Müller-Durovic et al. 1979).

The disease, therefore, can be viewed as a state of pathological metabolic programming superimposed upon a multifactorial pathogenic landscape. This unified view reframes MS not as a disease caused by metabolism alone, but as a disease in which metabolic reprogramming acts as a central amplifying mechanism and a modifiable therapeutic node, a concept that transforms our understanding of disease progression and opens up new possibilities for intervention. The stage is now set to apply this lens directly to the cellular players in MS pathogenesis.

## The immuno-metabolic axis in MS pathogenesis

### Connecting metabolism to pathology

Now that we have established the fundamental concepts of immunometabolism, we can apply this perspective to examine the complex pathophysiology of MS. The immune repertoire in MS is not a static group of cells; rather, it functions as a dynamic system that interacts with a microenvironment capable of altering its metabolic state and, consequently, its function. The key thesis from this section is that a profound and widespread dysregulation of cellular metabolism is a driving force behind the pathogenesis of MS, beginning with the initial activation of peripheral cells and extending to chronic, smoldering neuroinflammation (Wang et al. 2024). By examining the metabolic profiles of the principal cellular actors, we can start to comprehend MS not only as an immunological disorder but also as a systemic breakdown of metabolic homeostasis.

### Pro-inflammatory T cells (Th1 and Th17): the glycolytic drivers

Autoreactive CD4 + T cells, particularly those polarized toward the Th1 and Th17 lineages, are central to the initiation and propagation of CNS inflammation. These cells exhibit a metabolic profile that is fundamentally geared for rapid proliferation and effector function. Upon encountering myelin antigens in the periphery, these cells undergo a rapid and profound metabolic switch to aerobic glycolysis, driven by the mTOR-HIF-1α axis described previously. This glycolytic dependency is not merely a correlate of activation but a functional necessity (Meitei et al. 2026).

Studies have demonstrated that inhibiting glycolysis with 2-deoxyglucose (2-DG) or targeting glutaminolysis can effectively abrogate Th17 differentiation and ameliorate disease in experimental autoimmune encephalomyelitis (EAE), the animal model of MS. Th17 cells, in particular, have a unique metabolic addiction to glutamine and engage in a specific pathway of de novo FAS to generate pathogenic lipids. This heightened metabolic activity also makes them susceptible to distinct forms of cell death, such as ferroptosis, an iron-dependent form of cell death linked to lipid peroxidation. The inflammatory milieu within an MS lesion rich in cytokines like IL-23 and IL-1β serves to reinforce this glycolytic program, creating a vicious cycle of activation and inflammation (Hu et al. 2025; Lai et al. 2025; Mosure and Solt 2021).

#### Regulatory T cells (Tregs): the metabolically compromised peacekeepers

In a healthy immune system, the pro-inflammatory activity of effector T cells is kept in check by immunosuppressive Tregs. Tregs are metabolically distinct from their pro-inflammatory counterparts; they rely primarily on FAO and OXPHOS for their energy needs, a profile that supports their long-lived, suppressive phenotype (Lu et al. 2025; Getachew et al. 2025). However, MS profoundly compromises this metabolic stability. The inflammatory environment, characterized by high levels of pro-inflammatory cytokines and an abundance of glycolytic metabolites, actively destabilizes Tregs. They can undergo metabolic reprogramming, shifting away from FAO and towards glycolysis, which is the process of breaking down glucose for energy. This ‘‘metabolic plasticity’’ is detrimental, as it undermines their suppressive function and can even lead to their conversion into pro-inflammatory, IL-17-producing ex-Tregs (Lu et al. 2025; Kaushik and Yong 2021).

Furthermore, the metabolic competition within the inflammatory niche is substantial; rapidly proliferating, glycolytic effector T cells consume large quantities of glucose, potentially limiting the availability of resources required by Tregs to maintain their oxidative metabolism. This metabolic competition represents a crucial factor in the progression of autoimmune inflammation (Valentini et al. 2025).

### B cells: beyond antibodies to metabolic effectors

The success of B-cell depleting therapies (e.g., anti-CD20 antibodies like ocrelizumab and rituximab) in MS has refocused attention on the role of B lymphocytes. Beyond their well-known function as antibody-producing cells, B cells are potent antigen-presenting cells and sources of both pro-inflammatory (e.g., IL-6, GM-CSF) and anti-inflammatory (e.g., IL-10) cytokines (Sabatino et al. 2019; Nissimov et al. 2020). These distinct effector functions are, like those of T cells, governed by distinct metabolic programs. Pro-inflammatory B cells (effector B cells) tend to rely on glycolysis, while regulatory B cells (Bregs) that produce IL-10 are more dependent on FAO and OXPHOS. In patients with multiple sclerosis (MS), there is evidence of a metabolic imbalance in the B-cell compartment, characterized by a transition to a more glycolytic and pro-inflammatory phenotype (Wei et al. 2025; Iperi et al. 2021).

Furthermore, the metabolism of B cells within the CNS, particularly in the meninges where they can form tertiary lymphoid structures in progressive MS, likely contributes to the compartmentalized, smoldering inflammation that drives cortical demyelination and axonal loss (Pikor et al. 2016). Targeting the metabolic wiring of B cells, for instance by promoting a regulatory metabolic profile, could represent a novel therapeutic strategy that complements simple depletion, potentially leading to reduced inflammation and improved outcomes in MS patients (Boutitah-Benyaich et al. 2025; Sellebjerg and Weber 2021).

### Microglia and macrophages: The M1/M2 paradigm and beyond

Microglia, the resident innate immune cells of the CNS, and monocyte-derived macrophages infiltrating from the periphery are exquisitely sensitive to their microenvironment. They exhibit a remarkable degree of functional and metabolic plasticity. Classically activated, pro-inflammatory (often termed M1-like) microglia and macrophages rely heavily on glycolysis and a broken TCA cycle, which leads to the accumulation of succinate and itaconate (Ross et al. 2024). This glycolytic switch supports their production of ROS, nitric oxide (NO), and inflammatory cytokines. In contrast, alternatively activated pro-repair (M2-like) cells utilize FAO and OXPHOS to support their functions in tissue remodeling, debris clearance, and repair; (Engskog-Vlachos et al. 2025). However, in the chronically demyelinated cortex and normal-appearing white matter of progressive MS patients, microglia adopt a chronically activated, subtly inflamed phenotype (Blenkle et al. 2026).

This state, sometimes described as microglial paralysis or senescence, is characterized by metabolic dysfunction, including mitochondrial impairment and a shift away from efficient OXPHOS. These metabolically compromised cells are unable to support remyelination and instead contribute to a hostile environment that actively inhibits repair. The chronic inflammatory milieu likely locks these cells into a dysfunctional metabolic state from which they cannot easily escape (Sadeghdoust et al. 2024; Li et al. 2025b).

### Neurons: bioenergetic demands and vulnerability in inflammation

Neurons have exceptionally high energy demands, consuming substantial amounts of ATP to maintain ion gradients and support synaptic transmission (Rumpf et al. 2023). They are heavily dependent on mitochondrial function and oxidative phosphorylation, as well as metabolic support from nearby astrocytes through the astrocyte-neuron lactate shuttle. In the inflammatory environment of MS lesion, neurons encounter a dual metabolic threat. Direct inflammatory damage can impair mitochondrial function, resulting in energy deficits and increasing their susceptibility to excitotoxicity. Furthermore, the large number of activated, glycolytic immune cells consumes glucose and oxygen, which are critical for neuronal function. This bioenergetic crisis significantly contributes to axonal degeneration and leads to irreversible disabilities (Robinson et al. 2019; Carvalho Troitiño et al. 2025).

#### Oligodendrocyte lineage cells: starved of repair capacity

Oligodendrocytes, the myelin-producing cells of the CNS, and their precursors OPCs are also exquisitely sensitive to metabolic stress. Remyelination, the natural repair process, requires OPCs to proliferate, migrate to demyelinated axons, and differentiate into mature, myelinating oligodendrocytes (Sun et al. 2025b).

**Fig. 2:**
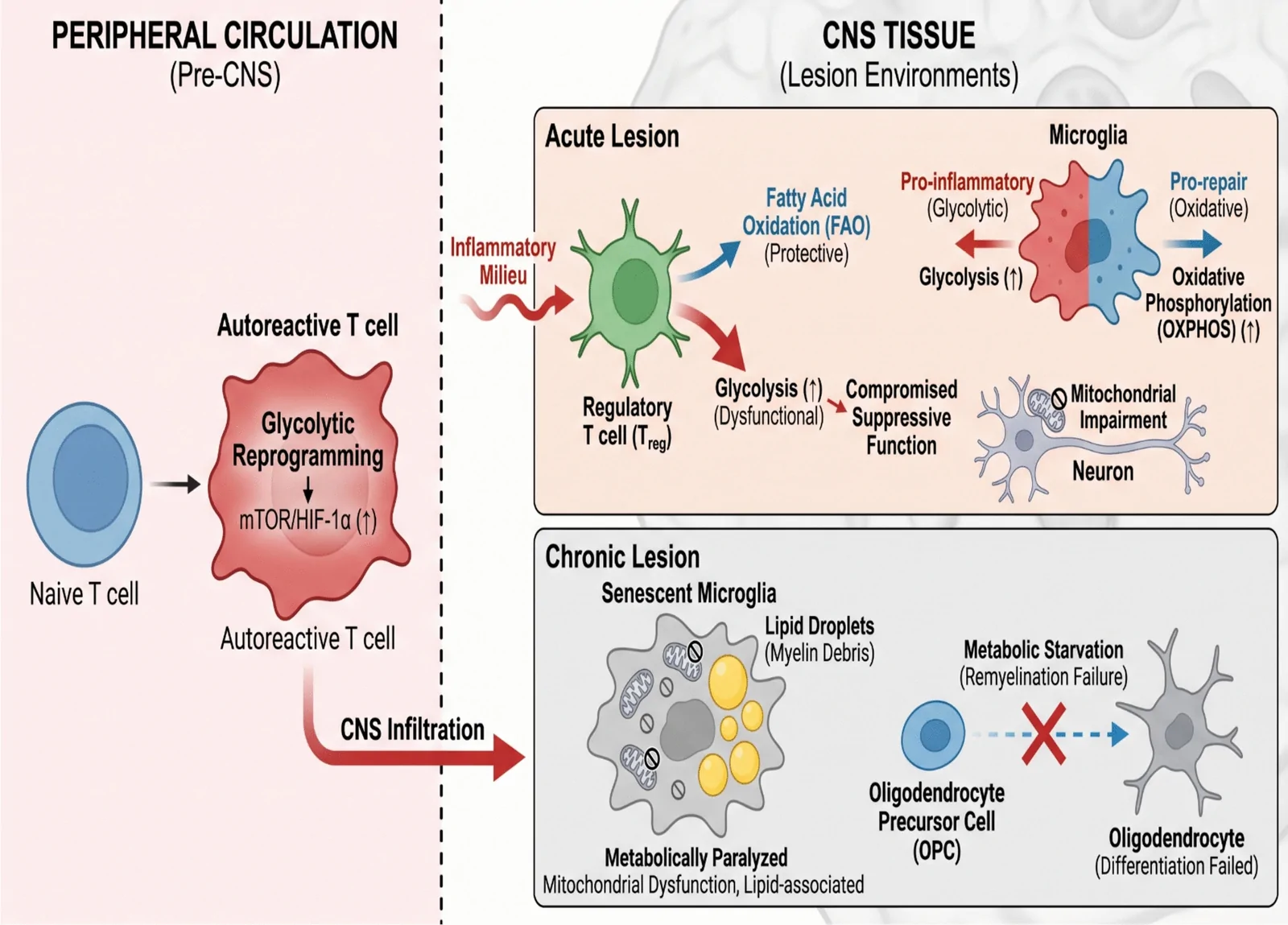
Cell-specific metabolic dysfunction drives MS pathogenesis. In the peripheral circulation, autoreactive T cells undergo glycolytic reprogramming via mTOR/HIF-1α, enabling CNS infiltration. Within CNS lesions, regulatory T cells (Tregs) shift from protective fatty acid oxidation (FAO) to glycolysis, compromising their suppressive function. Microglia exhibit phenotypic plasticity: acute lesions contain pro-inflammatory (glycolytic) and pro-repair (oxidative) populations, while chronic lesions feature metabolically paralyzed microglia with mitochondrial dysfunction and lipid droplet accumulation driven by myelin debris. Concurrently, neurons suffer mitochondrial impairment, leading to neurodegeneration and remyelination failure

Each of these steps is bioenergetically demanding. Differentiation, in particular, requires a metabolic shift and robust mitochondrial function. The inflammatory, metabolically depleted environment of the chronic MS lesion, characterized by hypoxia-like conditions, oxidative stress, and competition for nutrients, starves OPCs of the energy they need to complete this program. They may proliferate or migrate, but they fail to differentiate, leaving axons persistently demyelinated and vulnerable. Promoting the metabolic fitness of OPCs, for example by enhancing mitochondrial function or providing alternative fuels, is therefore a promising strategy to overcome remyelination failure (Zveik et al. 2024; Soung et al. 2025) **[**Fig. 2**]**.

### A unifying metabolic framework

The pathogenic landscape of MS is a complicated relationship among distinct but interconnected metabolic factors. The disease is characterized by a pathogenic imbalance in which glycolytic, pro-inflammatory lymphocytes and myeloid cells dominate, while regulatory T cells, oligodendrocyte precursor cells, and neurons exhibit metabolic impairment and deficiency in essential resources (Noroozi et al. 2025). This unified perspective redefines MS as a condition characterized by pathological metabolic programming across various cell types, paving the way for the next crucial inquiry: how do our existing therapies interact with this axis, and how can we deliberately target it for therapeutic benefit? Table 1 provides a detailed overview of the different metabolic processes that contribute to MS development, highlighting the metabolic patterns, important pathways, main regulators, and effects for each major cell type involved in the disease.

**Table 1:** Metabolic signatures of key cellular players in MS pathogenesis

Cell type	Metabolic program	Key pathways	Master regulators	Functional consequence in MS	Evidence level	Refs
Th1/Th17 Cells	Pro-inflammatory glycolytic	Aerobic glycolysis, glutaminolysis, FAS	mTOR↑, HIF-1α↑, AMPK↓	Rapid clonal expansion, CNS infiltration, production of inflammatory cytokines (IFN-γ, IL-17)	Human MS observational; EAE; in vitro	Shi et al. 2025; Wang et al. 2025b)
Regulatory T Cells (Tregs)	Anti-inflammatory oxidative	FAO, OXPHOS	AMPK↑, mTOR↓	Immune suppression, maintaining self-tolerance; compromised in MS due to metabolic destabilization	Human MS observational; EAE; in vitro	Lu et al. 2025; Kim et al. 2025)
B Cells (Effector)	Pro-inflammatory glycolytic	Aerobic glycolysis	mTOR↑	Antigen presentation, pro-inflammatory cytokine production (IL-6, GM-CSF)	Human MS observational; in vitro	Li et al. 2023; Zeng et al. 2020)
Regulatory B Cells (Bregs)	Anti-inflammatory oxidative	FAO, OXPHOS	AMPK↑	IL-10 production, immune regulation; functionally impaired in MS	Human MS observational; in vitro	Zhu et al. 2022; Varghese et al. 2024)
M1-like Microglia/Macrophages	Pro-inflammatory glycolytic	Aerobic glycolysis, broken TCA cycle (succinate accumulation)	HIF-1α↑, mTOR↑	Production of ROS, NO, inflammatory cytokines; tissue damage	Human MS observational; EAE; in vitro	He et al. 2024; Sun et al. 2024)
M2-like Microglia/Macrophages	Pro-repair oxidative	FAO, OXPHOS	AMPK↑	Tissue remodeling, debris clearance, repair promotion; impaired in chronic lesions	Human MS observational; EAE; in vitro	Sadeghdoust et al. 2024; Devanney et al. 2020)
Neurons	High-energy oxidative	OXPHOS, mitochondrial respiration	AMPK, SIRT1	Vulnerable to energy deficit in inflammatory milieu; mitochondrial impairment leads to axonal degeneration	Human MS observational; EAE; in vitro; mechanistic hypothesis	Ma et al. 2025; Chen et al. 2025)
Oligodendrocyte Precursor Cells (OPCs)	Differentiation-dependent	OXPHOS (for differentiation)	AMPK, mTOR (balanced)	Require metabolic fitness for proliferation, migration, and differentiation; starved in chronic lesions → remyelination failure	EAE/cuprizone; in vitro; mechanistic hypothesis	Sun et al. 2025b; Narine and Colognato 2022)

*Evidence Level Key*: Human MS RCT = evidence from randomized controlled trials in MS patients; Human MS observational/biomarker study = evidence from human MS cohort, biopsy, or biomarker studies; EAE/cuprizone = evidence from animal models of MS/demyelination; in vitro/ex vivo = evidence from cultured cells or ex vivo human samples; extrapolated evidence = evidence from other diseases (oncology, diabetes, aging) or general immunology; mechanistic hypothesis = proposed mechanism based on integrated evidence.

### Metabolic plasticity and heterogeneity beyond simple dichotomies

The preceding sections have utilized established frameworks, Th17/glycolysis versus Treg/FAO and M1/glycolysis versus M2/OXPHOS, to illustrate the immuno-metabolic axis in MS. While these dichotomies provide valuable conceptual clarity, they represent simplifications requiring qualification in a specialist context. T cell metabolic profiles are dynamic rather than fixed: under inflammatory conditions, Tregs can shift toward glycolysis, acquiring pro-inflammatory characteristics and losing suppressive capacity (Valentini et al. 2025), while Th17 cells can adopt oxidative metabolism, with pathogenic Th17 cells in MS exhibiting a hybrid metabolic state utilizing both glycolysis and OXPHOS (Soung et al. 2025). Similarly, the B-cell compartment displays substantial metabolic heterogeneity beyond the effector/Breg distinction, with naive, germinal center, memory, and plasma B cells each possessing distinct profiles, and metabolic reprogramming varying by anatomical location and activation status (Li et al. 2024).

Myeloid cell heterogeneity presents perhaps the most significant challenge to binary frameworks, as the M1/M2 paradigm, derived from in vitro studies, fails to capture the diversity of microglial and macrophage states observed in vivo (Jing et al. 2024; Ng et al. 2023). In MS lesions, myeloid cells display a spectrum of phenotypes with simultaneous expression of ‘‘classical’’ and ‘‘alternative’’ markers, and single-cell RNA sequencing has identified multiple distinct subpopulations, including disease-associated microglia, lipid-associated macrophages, and interferon-responsive cells, each with unique metabolic signatures (Lerma-Martin et al. 2024). The distinction between resident microglia and infiltrating monocyte-derived macrophages further complicates this picture, as these populations exhibit different metabolic dependencies and functional responses (De Vlaminck et al. 2022). The recognition of metabolic plasticity and heterogeneity has significant therapeutic implications: pharmacological interventions aimed at ‘‘locking’’ cells into a particular metabolic state may be undermined by adaptive rewiring, necessitating combination approaches, disease stage- and cell type-specific targeting, and biomarkers capable of tracking metabolic states to enable precision immuno-metabolic strategies.

### Pathological specificity of progressive MS and remyelination failure

The transition from RRMS to SPMS, as well as the clinical course of PPMS, is characterized by distinct pathological features that extend beyond acute inflammatory demyelination. Chronic active (paramagnetic rim) lesions (PRLs) represent a subgroup of white matter lesions with persistent inflammatory activity, characterized by iron-laden macrophages and microglia at the lesion edge. These PRLs are associated with more rapid clinical progression and are refractory to current B-cell-depleting therapies, indicating compartmentalized inflammation that persists for years. Concurrently, meningeal inflammation drives cortical grey matter pathology through lymphoid neogenesis in approximately 40% of progressive MS cases, creating a "surface-in" gradient of subpial demyelination and neuronal loss. The metabolic demands of chronically inflamed meningeal tissue and the resulting cortical damage contribute substantially to the bioenergetic crisis that defines progressive disease (Bagnato et al. 2024; Preziosa et al. 2026; Klotz et al. 2024).

Iron dysregulation and mitochondrial injury represent interconnected drivers of neurodegeneration in progressive MS. Iron builds up in myeloid cells at the edges of PRL, while oligodendrocytes in areas with myelin lose iron, leading to a harmful imbalance that increases oxidative stress. Ferroptosis, iron-dependent lipid peroxidation-driven cell death, has been implicated in oligodendrocyte loss and neuroinflammation, with PRL identification now included in the McDonald criteria for MS diagnosis (García-Salas et al. 2025; Nafe and Hattingen 2026; Fawzy et al. 2025, 2026a, 2026b; Fawzy and Fathy 2026).

Simultaneously, neuroinflammatory lesions cause widespread and persisting axonal ATP deficiency due to impaired electron transport chain function and depletion of TCA cycle enzymes in neuronal mitochondria. This axonal energy deficiency is initiated in acute lesions and persists chronically, providing a mechanistic link between early relapsing and later progressive pathology. The elevated extra-mitochondrial glucose metabolism observed in MS patients may represent a compensatory response to this mitochondrial dysfunction (Tai et al. 2023; Dutta et al. 2006).

Glial dysfunction and remyelination failure complete the pathological picture, with OPCs exquisitely sensitive to metabolic stress. OPCs exhibit decreased iron handling capabilities under inflammatory stimulation, and the metabolic demands of OPC differentiation, requiring a shift to oxidative phosphorylation and robust mitochondrial function, are not met in the inflammatory, metabolically depleted environment of chronic MS lesions, creating a persistent differentiation block (Zveik et al. 2024; Wu et al. 2025a). Disease stage-specific metabolic signatures further distinguish progressive forms: SPMS patients show elevated mitochondrial and peroxisomal stress metabolites, while PPMS patients exhibit profiles distinct from both RRMS and SPMS, with progressive MS characterized by marked impairment in energy metabolism pathways. These findings support a continuum model of MS pathophysiology while acknowledging that metabolic interventions must be stratified by disease stage, targeting glycolytic dependence in RRMS while emphasizing AMPK activation, NAD⁺ precursor supplementation, and iron metabolism modulation in progressive disease (Smusz et al. 2025; Senanayake et al. 2015; Oppong et al. 2024).

## Pharmacological re-interpretation of current MS therapies

### The metabolic lens on established therapeutics

The remarkable clinical success of disease-modifying therapies (DMTs) in relapsing–remitting MS has traditionally been attributed to their canonical immunological mechanisms: sequestration of lymphocytes, depletion of pathogenic cell populations, or blockade of trafficking molecules. However, this conventional narrative overlooks a crucial dimension of their pharmacology. As we learn more about immunometabolism, it becomes clear that many treatments have significant, and frequently direct, impacts on cellular metabolic pathways (Boutitah-Benyaich et al. 2025; Finocchiaro et al. 2025).

These metabolic effects discussed are not merely incidental; in several instances, they seem to be essential to therapeutic efficacy. This section will revisit the primary categories of MS therapies through the immuno-metabolic perspective established in the previous sections. By doing so, we aim to illustrate that the distinctions between ‘‘immunomodulator’’ and ‘‘metabolic modulator’’ are more fluid than previously understood and that comprehending these dual mechanisms can lead to more effective combination strategies and the advancement of next-generation therapeutics.

### Interferon-beta: linking inflammation to metabolic stress

IFN-beta is a type I interferon with pleiotropic immunomodulatory effects, including inhibition of Th17 cell differentiation, promotion of Treg function, and enhancement of blood–brain barrier integrity. From a metabolic perspective, IFN-beta signaling is intimately connected to cellular energy homeostasis (Khomich et al. 2025; Leisching et al. 2024). Engagement of the type I interferon receptor activates downstream signaling pathways, including JAK-STAT and PI3K-mTOR, which directly influence cellular metabolism. IFN-beta has been shown to induce the expression of genes involved in mitochondrial function and to modulate cellular redox state, which can enhance energy production and improve cellular responses to stress (Lin et al. 2025; Mazewski et al. 2020).

In the context of MS, IFN-beta may have therapeutic effects by modifying the metabolic fitness of pathogenic lymphocytes, potentially rendering them more susceptible to apoptosis or less capable of sustaining the high glycolytic flux required for their effector functions (Rizzo et al. 2016; Filipi and Jack 2020). Furthermore, IFN-beta's well-documented antiviral effects are themselves metabolically demanding, requiring significant mitochondrial respiration to mount an effective interferon-stimulated gene (ISG) response, which may indirectly compete with the metabolic resources available for inflammatory activation (Zhong et al. 2025; Ding et al. 2026).

#### Glatiramer acetate: promoting metabolic tolerance

Glatiramer acetate (GA), a random copolymer of four amino acids, is thought to exert its therapeutic effects by inducing GA-specific Tregs that migrate to the CNS and exert bystander suppression through the secretion of anti-inflammatory cytokines like IL-10 and TGF-beta (Schrempf and Ziemssen 2007). This induction of a regulatory T-cell phenotype is fundamentally a metabolic reprogramming event. As established in Sect. ‘‘The emerging field of immuno-metabolism: foundational concepts’’, Tregs are metabolically distinct from effector T cells, relying on FAO and OXPHOS rather than glycolysis. The differentiation of naive T cells into GA-specific Tregs therefore requires a metabolic switch, likely orchestrated by AMPK activation and mTOR inhibition (Lu et al. 2025; Getachew et al. 2025). By promoting an immunological environment conducive to Treg generation, GA may be indirectly favoring a metabolic landscape characterized by oxidative metabolism and immune tolerance. This perspective suggests that the therapeutic efficacy of GA may be partially dependent on successful metabolic reprogramming of the T-cell compartment (Shi et al. 2025; Qiu et al. 2025). While the induction of GA-specific Tregs is well-established (Spadaro et al. 2017), the degree to which this phenomenon reflects direct metabolic modulation versus indirect consequences of altered cytokine signaling remains an open hypothesis requiring further mechanistic investigation.

### Sphingosine-1-phosphate receptor modulators: beyond lymphocyte retention

Fingolimod, the first oral DMT for MS, and its next-generation counterparts siponimod and ozanimod, are widely appreciated for their ability to trap lymphocytes in lymph nodes by downmodulating sphingosine-1-phosphate (S1P) receptors. However, S1P receptors are ubiquitously expressed throughout the body, including on cells of the CNS, and their signaling is inextricably linked to cellular metabolism (Kihara and Chun 2023).

#### Direct CNS metabolic effects

S1P signaling regulates numerous cellular processes beyond migration, including proliferation, survival, and metabolism. In neural cells, S1P receptor engagement modulates mitochondrial function, autophagy, and cellular stress responses. Siponimod, which is approved for active secondary progressive MS, has been shown to have direct neuroprotective and remyelination-promoting effects in preclinical models. These effects are not related to changes in the immune system outside of the brain. These central effects are likely mediated, at least in part, through metabolic mechanisms (Binish and Xiao 2025; Shen et al. 2014).

S1P receptor signaling can activate the PI3K-Akt-mTOR pathway, influencing cellular growth and metabolism, and it can also modulate sphingolipid metabolism itself, with downstream consequences for membrane composition and cell signaling. By penetrating the CNS and engaging S1P receptors on astrocytes, neurons, and oligodendrocytes, these drugs may be directly influencing the metabolic health of the CNS parenchyma, promoting mitochondrial fitness, and supporting cellular resilience in the face of inflammatory stress (Powell and Pitson 2025; Alobid 2026).

### Natalizumab: metabolic consequences of compartmentalization

Natalizumab, a monoclonal antibody against α4-integrin, potently blocks the migration of lymphocytes across the blood–brain barrier by interfering with their adhesion to VCAM-1 on endothelial cells. Its primary mechanism is therefore anatomical: it prevents pathogenic immune cells from reaching their target organ. However, this sequestration has profound metabolic consequences. Lymphocytes that cannot enter the CNS are kept in the peripheral compartment, which has a very different metabolic environment than the inflamed CNS niche (Nguyen Ky et al. 2023; Khoy et al. 2020).

The peripheral lymphoid organs, characterized by distinct nutrient availability, oxygen tension, and cellular interactions, impose a unique metabolic program on the lymphocytes trapped within them. Natalizumab further alters the metabolic environment of the CNS by reducing the influx of new, activated, glycolytic immune cells. This reduction decreases the metabolic pressure on resident cells and may facilitate a recovery of their metabolic function.

The significant resurgence in disease activity after the cessation of natalizumab treatment is believed to be partly attributable to the abrupt reintroduction of a metabolically primed, highly pathogenic lymphocyte reservoir into circulation. This may lead to their swift penetration into a metabolically susceptible CNS (Kivisäkk et al. 2009; Koudriavtseva et al. 2014). This interpretation remains speculative, as direct evidence connecting rebound pathology to the pre-existing metabolic priming of sequestered lymphocytes is scarce. Alternative explanations, including the re-expansion of autoreactive clones and the disruption of blood–brain barrier integrity, are also credible and not mutually exclusive.

### Nrf2 activation and beyond

The primary mechanism by which Dimethyl fumarate (DMF) operates is to activate the Nrf2 pathway. DMF modifies Kelch-like ECH-associated protein 1 (Keap1) to enhance the stability of Nrf2, facilitating its translocation to the nucleus and boosting the transcription of various genes that provide cellular protection and function as antioxidants. Nrf2 regulates the enzymes responsible for synthesizing glutathione, NADPH (via the pentose phosphate pathway), and mitochondrial function, which is inherently metabolic (Zingkou et al. 2026; Abu-Halaka et al. 2025).

By activating Nrf2, DMF fundamentally rewires the cellular metabolic state towards resilience against oxidative stress, a hallmark of the inflammatory MS lesion. This process has direct consequences for both immune cells and CNS-resident cells. In myeloid cells, DMF promotes a shift away from a pro-inflammatory, glycolytic phenotype. In T cells, it can inhibit Th1 and Th17 differentiation while favoring Treg generation, a functional outcome that is entirely consistent with a metabolic shift towards oxidative metabolism (Tonev and Momchilova 2023; Manai and Amadio 2022). Thus, DMF can be understood as a drug that pharmacologically enforces an anti-inflammatory metabolic program across multiple cell types, aligning perfectly with the concept of immuno-metabolic reprogramming.

### Teriflunomide: direct metabolic targeting

If DMF acts as a broad metabolic modulator, teriflunomide exemplifies more direct metabolic targeting. Teriflunomide selectively and reversibly inhibits dihydroorotate dehydrogenase (DHODH), a mitochondrial enzyme critical for the de novo synthesis of pyrimidines. Rapidly proliferating lymphocytes, such as those expanding in response to myelin antigens, are heavily dependent on de novo pyrimidine synthesis to generate the nucleotides required for DNA replication. In contrast, resting lymphocytes and other cell types can rely on salvage pathways to meet their pyrimidine needs (Li et al. 2021; Yang et al. 2024).

By starving dividing lymphocytes of the building blocks for DNA synthesis, teriflunomide selectively curtails their clonal expansion without broadly killing all immune cells. This is a textbook example of targeting a metabolic vulnerability specific to a pathogenic cell state. The mechanism underscores a core principle of immuno-metabolic pharmacology: understanding the unique metabolic dependencies of pathogenic cells allows for the design of therapies with a wide therapeutic window (Wu et al. 2023; Mirzapoiazova et al. 2024).

### BTK inhibitors: non-depleting metabolic correctors of B-cell and myeloid dysfunction

Bruton’s tyrosine kinase (BTK) inhibitors constitute the newest class of disease-modifying therapies to enter late-stage clinical development for MS, with several agents, including evobrutinib, tolebrutinib, fenebrutinib, and remibrutinib, having advanced through Phase II–III trials. Unlike anti-CD20 monoclonal antibodies that deplete B cells, BTK inhibitors are oral, brain-penetrant small molecules that modulate BTK signaling in both B cells and myeloid cells without eliminating them. This mechanistic distinction has profound immuno-metabolic implications that align precisely with the thesis of this review (Montalban et al. 2024; Airas et al. 2024; Trojano and Paolicelli 2025; Bar-Or et al. 2025).

The metabolic effects of BTK inhibition are most clearly established in the B-cell compartment. Pro-inflammatory effector B cells in MS patients exhibit a dependence on elevated OXPHOS compared to anti-inflammatory, IL-10-producing regulatory B cells. BTK inhibition directly curtails B-cell mitochondrial respiration but not glycolysis, thereby limiting their capacity to serve as antigen-presenting cells to T cells and attenuating their activation-induced expression of costimulatory molecules (Li et al. 2022).

This metabolic modulation produces an anti-inflammatory shift in the B-cell cytokine profile, reducing the GM-CSF/IL-10 ratio and reversing the B-cell cytokine imbalance characteristic of MS. Notably, these effects are achieved without B-cell depletion; rather, BTK inhibitors “correct the metabolic abnormality” of pathogenic B cells, rendering them less capable of driving pro-inflammatory responses in T cells and myeloid cells. Phase 1 studies in healthy volunteers have confirmed that in vivo BTK treatment reduces circulating B-cell mitochondrial respiration and mediates an anti-inflammatory shift in B-cell responses associated with attenuated T-cell pro-inflammatory responses (Li et al. 2024; Krämer et al. 2023).

Beyond B cells, BTK is expressed in myeloid cells, including macrophages and microglia, where its inhibition exerts additional metabolic effects. In both murine and human myeloid cells, BTK inhibition with evobrutinib and tolebrutinib significantly decreases the oxygen consumption rate and extracellular acidification rate, indicating suppression of both OXPHOS and glycolytic flux. BTK signaling regulates macrophage polarization state by modulating mitochondrially encoded electron transport chain subunits, and its inhibition shifts macrophages from a pro-inflammatory M1-like state toward a pro-resolution M2-like phenotype by promoting OXPHOS. In myeloid cells, BTK inhibition also alters microRNA expression profiles, decreasing pro-inflammatory miR-155-5p and increasing miR-223-3p, consistent with a dampened inflammatory phenotype (Purvis et al. 2024; Geladaris et al. 2024).

The brain-penetrant nature of second-generation BTK inhibitors, particularly tolebrutinib and fenebrutinib, enables targeting of compartmentalized neuroinflammation within the CNS, a key driver of progressive MS. Microglia, the resident innate immune cells of the brain, express BTK, and fenebrutinib has been shown to block deleterious microglial Fcγ receptor activation, including cytokine and chemokine release, microglial clustering, and neurite damage in human brain cell systems. Gene expression analyses have identified pathways linked to inflammation and cholesterol metabolism that are modulated by fenebrutinib treatment. BTK inhibition also dampens pro-inflammatory microglial activity stimulated by immune complexes and may reduce iron import and storage in activated microglia and macrophages within chronic active MS lesions (Turner et al. 2024; Lambe and Fox 2026; Reich et al. 2021; Gruber et al. 2024).

Preliminary evidence further suggests that BTK inhibitors may alleviate oxidative stress and promote remyelination, possibly through their impact on microglia. By modulating the metabolic and inflammatory state of both peripheral immune cells and CNS-resident microglia, BTK inhibitors offer a uniquely integrated approach to immuno-metabolic reprogramming that spans the peripheral and central compartments. This positions them as potentially transformative for progressive MS, where compartmentalized smoldering inflammation and remyelination failure remain major therapeutic challenges (Geladaris et al. 2024; Gruber et al. 2024; Elkjaer et al. 2023).

In the context of the immuno-metabolic framework advanced in this review, BTK inhibitors can be understood as non-depleting metabolic correctors that restore homeostatic metabolic programming in B cells and myeloid cells without the immunosuppressive consequences of cell depletion. By reducing mitochondrial OXPHOS in pathogenic B cells and promoting a metabolic shift toward an M2-like state in myeloid cells, these agents exemplify the paradigm of targeted metabolic reprogramming, a strategy that holds particular promise for addressing the unmet needs of progressive MS.

### A unified metabolic view of DMTs

This reinterpretation suggests that metabolic modulation contributes to the therapeutic efficacy of several DMT classes, complementing their canonical immunological mechanisms. However, the relative contribution of metabolic versus immunological effects varies substantially between agents and remains incompletely characterized for most therapies. Teriflunomide inhibits specific enzymes, fumarates enhance Nrf2 activity, and other processes involve trapping and reducing cells. Almost every type of DMT affects cellular metabolism in some way.

This reinterpretation has profound implications. It confirms the immuno-metabolic axis's central role in MS and suggests that the clinical success of these treatments is, in part, a sign of how effective metabolic intervention can be. This understanding naturally points to the next step: deliberately focusing on metabolism to improve current treatments and meet the needs of progressive MS and remyelination failure, which require better options for helping repair nerves and enhancing patient results, such as developing new therapies that target metabolic pathways involved in nerve repair and regeneration. Table 2 provides a comprehensive summary of current MS disease-modifying therapies, contrasting their canonical immunological mechanisms with the emerging understanding of their immuno-metabolic effects and the clinical implications of this reinterpretation.

**Table 2:** Current MS therapies reinterpreted through a metabolic lens

Drug class	Representative agents	Canonical immune mechanism	Immuno-metabolic mechanism	Clinical implications	Evidence level and mechanism certainty	Refs
Interferons	Interferon-beta	Anti-inflammatory cytokine modulation	Induces mitochondrial gene expression, modulates cellular redox state, alters metabolic fitness of lymphocytes	Metabolic effects may contribute to therapeutic efficacy but are indirect	Human MS RCT; in vitro **Indirect** (downstream of cytokine signaling)	Khomich et al. 2025; Bellucci et al. 2023)
Amino Acid Copolymer	Glatiramer acetate	Induces GA-specific Tregs	Promotes Treg differentiation requiring metabolic switch (FAO/OXPHOS) via AMPK activation and mTOR inhibition	Efficacy depends on successful metabolic reprogramming of T-cell compartment	Human MS RCT; EAE; in vitro **Hypothesis** (mechanism inferred from Treg biology, not directly demonstrated for GA)	De Riccardis et al. 2016)
S1P Receptor Modulators	Fingolimod, Siponimod, Ozanimod	Lymphocyte sequestration in lymph nodes	Direct CNS effects: modulates mitochondrial function, autophagy, cellular stress responses in astrocytes, neurons, oligodendrocytes	Contributes to neuroprotection and remyelination in progressive MS	Human MS RCT; EAE; in vitro; mechanistic hypothesis **Direct** (supported by preclinical mechanistic studies)	Coyle et al. 2024; Dumitrescu et al. 2023)
Integrin Inhibitor	Natalizumab	Blocks lymphocyte trafficking across BBB	Indirect metabolic effects: retains lymphocytes in peripheral compartment with distinct metabolic environment; reduces metabolic competition in CNS	Rebound disease reflects release of metabolically primed pathogenic cells	Human MS RCT; human MS observational **Hypothesis** (metabolic priming of rebound lymphocytes is speculative; alternative explanations exist)	Nguyen Ky et al. 2023; Khoy et al. 2020)
Fumarates	Dimethyl fumarate	Anti-inflammatory, antioxidant	Activates Nrf2 pathway → upregulates antioxidant enzymes, glutathione synthesis, NADPH generation; promotes shift from glycolysis to OXPHOS	Paradigmatic example of metabolic modulation enforcing anti-inflammatory program	Human MS RCT; EAE; in vitro **Direct** (well-established Nrf2-dependent mechanism)	Oliveira 2026)
DHODH Inhibitor	Teriflunomide	Inhibits proliferating lymphocytes	Directly inhibits dihydroorotate dehydrogenase (DHODH), blocking de novo pyrimidine synthesis in rapidly dividing cells	Precision metabolic targeting exploiting vulnerability of pathogenic lymphocytes	Human MS RCT; in vitro; extrapolated from oncology **Direct** (enzymatic target well characterized)	Yang et al. 2024; Mullen et al. 2024; Tilly et al. 2021)
Anti-CD20 Antibodies	Rituximab, Ocrelizumab, Ofatumumab	B-cell depletion	Metabolic repopulation: immune reconstitution from precursors imposes metabolic stress on emerging autoreactive clones	Long-term remission may depend on metabolic constraints of immune repopulation	Human MS RCT; human MS observational **Hypothesis** (long-term efficacy mechanism speculative; requires longitudinal metabolic profiling)	Willison et al. 2025; Heming and Wiendl 2023)
Purine Analog	Cladribine	Lymphocyte depletion	Similar metabolic repopulation effect; rebuilding immune compartment requires massive metabolic activity	Dosing schedules could be optimized based on metabolic recovery	Human MS RCT; human MS observational **Hypothesis** (extrapolated from anti-CD20; limited direct evidence)	Cammarata et al. 2026)
BTK Inhibitors (Emerging)	Evobrutinib, Tolebrutinib, Fenebrutinib, Remibrutinib	B-cell and myeloid signaling modulation (non-depleting)	Curtains B-cell mitochondrial OXPHOS; shifts macrophages from M1-like to M2-like via OXPHOS promotion; modulates microglial function	Non-depleting metabolic correctors; potential for progressive MS	Human MS RCT; human MS observational **Direct** (supported by mechanistic studies in human cells and animal models)	Montalban et al. 2024; Airas et al. 2024; Trojano and Paolicelli 2025; Bar-Or et al. 2025; Geladaris et al. 2024; Gruber et al. 2024)

## Novel pharmacological strategies: targeting the axis

### From accidental to intentional targeting

The preceding section highlighted that many effective MS therapies engage metabolic pathways, often as an incidental aspect of their pharmacology. This insight supports the immuno-metabolic axis as a relevant target for pharmacological intervention. The next logical step is to transition from serendipitous findings to intentional design, aiming to develop therapies that specifically modulate cellular metabolism for therapeutic benefit. This represents an evolving direction in MS drug discovery.

We can now pose the question, ‘‘Which metabolic state should we induce?’’ rather than ‘‘Which immune cell should we kill or block?’’ This section examines the innovative pharmacological strategies that arise from this inquiry. We will examine direct metabolic enzyme inhibitors, modulators of master regulators, prospects for repurposing drugs from other disease domains, and the potential for dietary interventions to augment pharmacotherapy. Collectively, these approaches represent a new frontier in MS treatment, particularly promising for tackling the previously intractable challenges of progressive disease and remyelination failure.

### Glycolysis inhibitors: starving the inflammatory engine

Given the heavy reliance of pro-inflammatory Th1, Th17, and M1-like myeloid cells on aerobic glycolysis, direct glycolytic inhibition represents an attractive strategy. 2-DG, a glucose analog that inhibits hexokinase, the first enzyme in glycolysis, has demonstrated efficacy in preclinical EAE models, reducing Th17 cell frequency and attenuating disease severity. However, the relatively blunt nature of 2-DG and its potential for systemic toxicity limit its clinical translation, particularly due to the risk of affecting normal cellular metabolism and causing adverse effects in patients(Wang et al. 2025b; Cheng et al. 2021).

More sophisticated approaches are under development, including inhibitors of pyruvate kinase M2 (PKM2), the isoform of pyruvate kinase that is specifically upregulated in proliferating and inflammatory cells and regulates the metabolic switch to aerobic glycolysis. PKM2 activators, paradoxically, can disrupt the tetrameric structure required for their function in glycolysis, shifting cells toward OXPHOS (Das et al. 2025; Chen et al. 2024). Compounds like TEPP-46 have shown promise in preclinical models by dampening inflammatory responses. The challenge lies in achieving selectivity for pathogenic immune cells while sparing other glycolytic tissues, such as those in the brain and muscle, which are critical to maintaining normal physiological functions (Xia et al. 2025; Lin et al. 2024).

#### Glutaminase inhibitors: targeting Th17 metabolism

The distinct dependency of Th17 cells on glutaminolysis makes glutaminase inhibitors an especially promising therapeutic strategy (Liu et al. 2023; Kono et al. 2018). Glutaminase (GLS) is the first enzyme in glutamine metabolism, converting glutamine to glutamate (Jiang et al. 2025). CB-839 (telaglenastat), a potent and selective GLS1 inhibitor, has advanced to clinical trials in oncology (Jamshidi-Parsian et al. 2024). Preclinical studies in EAE have demonstrated that pharmacological inhibition of glutaminase or genetic ablation of GLS1 in T cells profoundly impairs Th17 differentiation and protects against neuroinflammation. By limiting the availability of glutamine-derived carbon and nitrogen required for Th17 cell proliferation and effector function, these agents can selectively disarm a key pathogenic population without broadly affecting all lymphocytes. Given the emerging role of B cells in MS, investigating the effects of glutaminase inhibition on pathogenic B-cell subsets is a logical next step (Sabatino et al. 2025; Hollinger et al. 2019).

#### Fatty acid metabolism modulators: balancing inflammation and regulation

Targeting the balance between FAS and FAO offers another avenue. Acetyl-CoA carboxylase 1 (ACC1) is the rate-limiting enzyme in de novo FAS and is essential for Th17 cell differentiation. Inhibitors of ACC1, such as ND-646 or Soraphen A, have shown efficacy in EAE by blocking the production of the specific phospholipids required for Th17 cell function (Young et al. 2017; Svensson et al. 2016; Berod et al. 2014).

Conversely, promoting FAO can enhance Treg stability and function. While direct FAO activators are less developed, approaches that indirectly boost FAO, such as AMPK activation, are further along, and these methods may provide a viable alternative for enhancing Treg function in the context of autoimmune diseases. The ideal but challenging pharmacological balancing act involves inhibiting FAS in pathogenic cells while promoting FAO in regulatory cells (Getachew et al. 2025; Härm et al. 2025).

### mTOR inhibitors: restoring immune tolerance

As the central node integrating nutrient and inflammatory signals, mTOR is an exceptionally attractive target. The prototypical mTOR inhibitor, rapamycin (sirolimus), and its analogs (rapalogs) have been extensively studied in autoimmune models. By inhibiting mTORC1, rapamycin suppresses the glycolytic burst required for effector T-cell differentiation while simultaneously promoting Treg generation and function (Sun et al. 2018; Geier and Perl 2021). In EAE, rapamycin administration ameliorates disease and promotes a regulatory immune environment. However, concerns about on-target toxicities, such as metabolic disturbances (hyperglycemia, hyperlipidemia) and immunosuppression, have hampered the clinical translation of systemic mTOR inhibition in MS (Li et al. 2020).

Strategies to overcome these limitations include the development of selective mTOR inhibitors that spare mTORC2, the use of nanoparticle formulations to target delivery to pathogenic cells, and the exploration of intermittent dosing regimens that achieve immunological effects while minimizing metabolic side effects (Yoon 2020; Tang et al. 2024; Borsari et al. 2021). The FDA approval of mTOR inhibitors for other indications (e.g., organ transplantation, lymphangioleiomyomatosis) provides a wealth of clinical safety data that could accelerate repurposing efforts in MS (Elia et al. 2025; Park and Lee 2023).

#### AMPK activators: the cellular energy guardians

mTOR is the accelerator of inflammation, while AMPK slows it down. Activating AMPK shifts cells away from anabolic, glycolytic growth and towards catabolic, oxidative metabolism, a state conducive to immune tolerance and cellular resilience. Metformin, a primary diabetes medication and AMPK activator, has attracted considerable interest as a potential candidate for repurposing in MS. Metformin influences peripheral glucose metabolism and traverses the BBB, exerting direct effects on cells within the CNS. Preclinical research has demonstrated that metformin can facilitate Treg differentiation, suppress Th17 responses, and notably, augment remyelination by encouraging OPC differentiation (Keersmaecker et al. 2024; Neumann et al. 2019; Sheng et al. 2026).

The mechanism of this pro-remyelination effect appears to involve AMPK-driven metabolic changes that overcome the differentiation block imposed by the inflammatory milieu. Several clinical trials are currently underway or planned for evaluating metformin in MS, either as a monotherapy or in combination with existing DMTs (Narine et al. 2023; Chen 2026). Other AMPK activators, such as AICAR, are also in preclinical development but face challenges related to bioavailability and specificity, which may hinder their effectiveness in treating conditions like MS, particularly in ensuring that they can effectively target the pathways necessary for remyelination without causing adverse effects (Moore et al. 2020; Višnjić et al. 2021).

#### HIF-1α inhibitors: disrupting the pseudo-hypoxic state

The stabilization of HIF-1α in inflammatory cells under normoxic conditions (pseudo-hypoxia) represents a key pathogenic mechanism in MS. Direct pharmacological inhibition of HIF-1α is challenging due to its role in physiological responses to hypoxia. However, strategies to disrupt its upstream stabilization or downstream transcriptional activity are emerging (Giordano et al. 2026; Hollingworth et al. 2025).

**Fig. 3:**
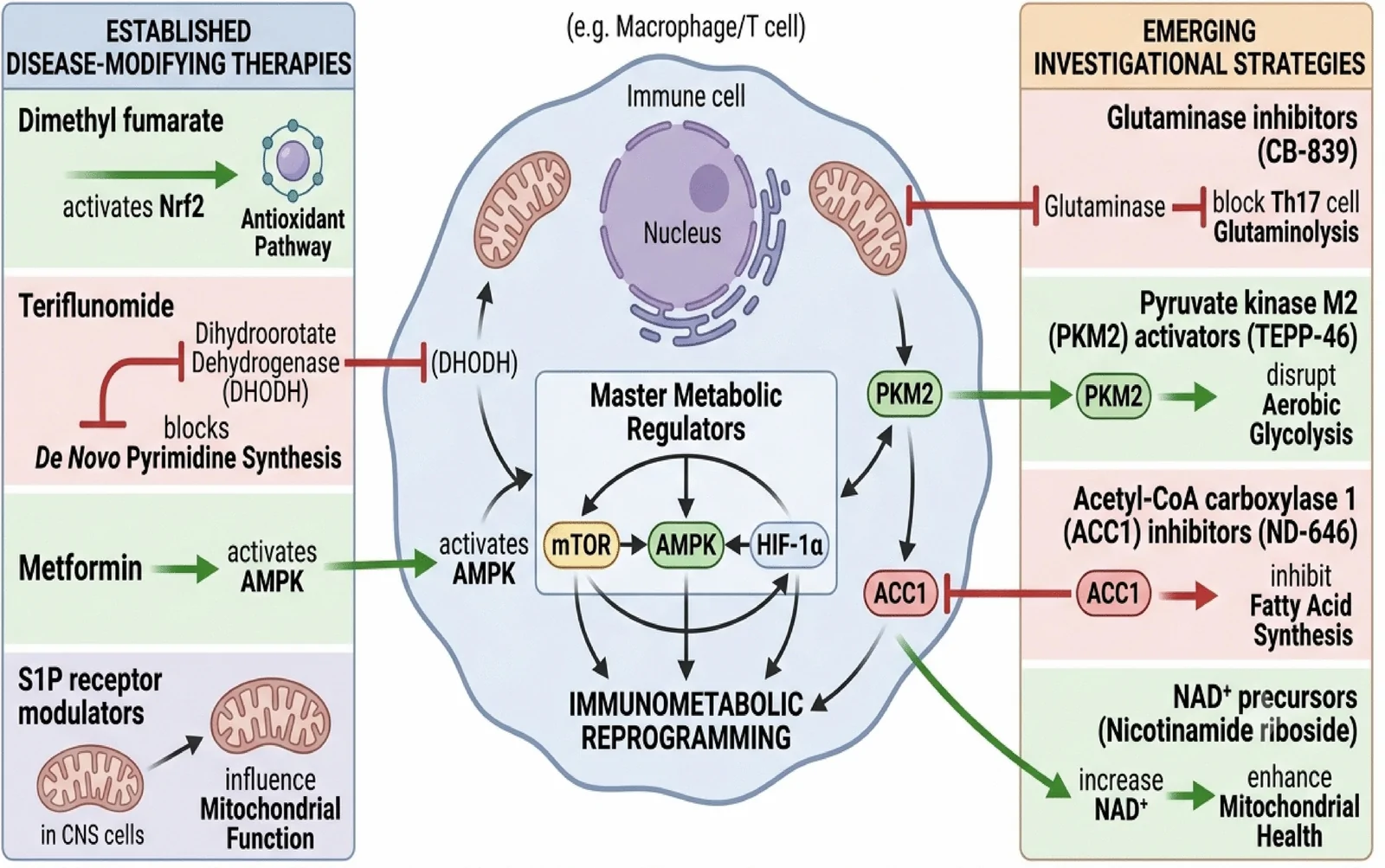
Pharmacological targeting of the immuno-metabolic axis. Established disease-modifying therapies exert direct metabolic effects: dimethyl fumarate activates the Nrf2 antioxidant pathway; teriflunomide inhibits dihydroorotate dehydrogenase (DHODH), blocking de novo pyrimidine synthesis; metformin activates AMPK; and sphingosine-1-phosphate (S1P) receptor modulators influence mitochondrial function in CNS cells. Emerging investigational strategies target specific metabolic vulnerabilities: glutaminase inhibitors (CB-839) block Th17 cell glutaminolysis; pyruvate kinase M2 (PKM2) activators (TEPP-46) disrupt aerobic glycolysis; acetyl-CoA carboxylase 1 (ACC1) inhibitors (ND-646) inhibit fatty acid synthesis; and NAD + precursors (nicotinamide riboside) enhance mitochondrial health. Master metabolic regulators, mTOR, AMPK, and HIF-1α, serve as central nodes for immunometabolic reprogramming

Digoxin and other cardiac glycosides have been shown to inhibit HIF-1α protein synthesis and have demonstrated efficacy in EAE by selectively suppressing Th17 differentiation (Huh et al. 2011; Zhang et al. 2008). Acriflavine, a drug with a long history of use as a topical antiseptic, binds to HIF-1α and inhibits its dimerization with HIF-1β, blocking its transcriptional activity (Piorecka et al. 2022). While these agents are not yet ready for clinical translation in MS due to toxicity and off-target effects, they validate HIF-1α as a therapeutic target and spur the development of more selective inhibitors **[**Fig. 3**]**.

### Repurposing opportunities: old drugs, new metabolic indications

The drug repurposing approach offers the advantage of known safety profiles, established manufacturing processes, and potentially faster pathways to clinical implementation. The immuno-metabolic axis is replete with repurposing opportunities (Zhang et al. 2026).

#### Metformin: the leading candidate

As discussed above, metformin is the most advanced repurposing candidate. Its well-characterized safety profile, low cost, and oral bioavailability make it an ideal candidate for rapid translation. The ongoing trials will determine whether the promising preclinical effects translate into clinical benefit for people with MS, particularly in the progressive phase where remyelination failure is a dominant pathology (Keersmaecker et al. 2024; Zhou and Xue 2025).

#### Statins: beyond cholesterol lowering

Statins, inhibitors of HMG-CoA reductase, are among the most widely prescribed drugs worldwide (Muhammad et al. 2026). Beyond their lipid-lowering effects, statins have pleiotropic immunomodulatory effects that are fundamentally metabolic. By inhibiting the mevalonate pathway, statins deplete intermediates necessary for the prenylation of small GTPases, such as Ras and Rho, which are crucial for intracellular signaling and metabolic regulation. This depletion results in the suppression of inflammatory gene expression and modulation of T-cell responses (Martin et al. 2001; Oesterle et al. 2017; Zarief 2025; Freuer et al. 2025). Although clinical trials of statins in MS have produced mixed results, further investigating their immunometabolic effects, including their influence on T-cell modulation and inflammatory gene expression, could enhance patient selection and inform combination treatment strategies (Abdalla et al. 2021; Chataway et al. 2025).

#### Nicotinamide riboside and NAD + precursors: boosting mitochondrial health

The age-related decline in NAD + (nicotinamide adenine dinucleotide) levels is implicated in metabolic dysfunction, mitochondrial impairment, and neuroinflammation. Nicotinamide riboside (NR) and nicotinamide mononucleotide (NMN) are NAD + precursors that have gained attention as nutritional supplements with potential therapeutic applications. In preclinical models, NR supplementation has been shown to enhance mitochondrial function, promote remyelination, and improve outcomes in EAE (Rosenkranz et al. 2021; Lapatto et al. 2023; Wu et al. 2025b). By boosting NAD + levels, these agents may enhance the activity of sirtuins (particularly SIRT1), which are NAD + -dependent deacetylases that regulate mitochondrial biogenesis and inflammation (Rosenkranz et al. 2021; Cerutti et al. 2014). While the evidence base is still evolving, NAD + precursors represent a safe and accessible intervention that could support metabolic health in the aging CNS of progressive MS patients.

#### Other repurposing candidates: pioglitazone, bezafibrate, and beyond

Pioglitazone, a PPAR-γ agonist used for diabetes, has shown anti-inflammatory and neuroprotective effects in preclinical MS models and small clinical trials. PPAR-γ is a nuclear receptor that regulates lipid metabolism and inflammation. Bezafibrate, a pan-PPAR agonist, has also demonstrated efficacy in EAE by promoting mitochondrial function and fatty acid oxidation. These agents, along with others like resveratrol (a SIRT1 activator) and trehalose (an inducer of autophagy), represent a growing pharmacopeia of metabolic modulators awaiting rigorous evaluation in MS (Wang et al. 2018; Bhargava 2021; Drew et al. 2008; Gray et al. 2012).

### Ketogenic diet and caloric restriction

The ketogenic diet, a high-fat, low-carbohydrate diet that shifts metabolism towards ketone body production and FAO, has garnered interest in MS (Brenton et al. 2022). Ketone bodies (β-hydroxybutyrate and acetoacetate) are not just alternative fuels; they are signaling molecules that can inhibit the NLRP3 inflammasome and modulate histone acetylation (García-Rodríguez and Giménez-Cassina 2021; Youm et al. 2015). Preclinical studies suggest that ketogenic diets can ameliorate EAE (Brockhoff et al. 2023). Similarly, caloric restriction and intermittent fasting activate AMPK and inhibit mTOR, promoting autophagy and cellular resilience (Vergara Nieto et al. 2025; Xinyan et al. 2025). These interventions may create a systemic metabolic environment that favors regulatory over inflammatory immune responses.

The gut microbiome constitutes a modifiable component within the immuno-metabolic axis. Commensal bacteria generate SCFAs, chiefly acetate, propionate, and butyrate, which act as energy sources for colonocytes and directly influence systemic immune function. SCFAs activate GPR41 and GPR43 receptors on T cells and macrophages, facilitating the differentiation of regulatory T cells and inhibiting the production of pro-inflammatory cytokines. Fecal metabolomic studies in multiple sclerosis patients demonstrate diminished short-chain fatty acid levels and modified microbial composition relative to healthy controls. Dietary interventions that augment SCFA production, such as high-fiber diets and probiotic supplementation, may synergize with pharmacologic metabolic reprogramming by establishing a systemic tolerogenic milieu. Conversely, gut dysbiosis may undermine the efficacy of metabolic drugs, indicating that microbiome status could affect patient stratification for immuno-metabolic therapies (Sharma 2025; Feng et al. 2025).

## Challenges, limitations, and future directions

### Clinical implementation challenges

Implementing the immuno-metabolic axis in clinical practice encounters three significant challenges. First, attaining cellular specificity without on-target toxicity is challenging due to the necessity of glycolysis and oxidative phosphorylation in both pathogenic and healthy cells. This necessitates the identification of genuine metabolic "Achilles' heels" that are uniquely vital to pathogenic cells, such as glutamine dependency in Th17 cells or particular lipid desaturation needs in inflammatory microglia. Second, immune cells demonstrate remarkable metabolic adaptability, enabling them to enhance alternative energy pathways and circumvent pharmacological inhibition, which may result in treatment resistance.

Furthermore, the stage of multiple sclerosis is significant: a glycolysis inhibitor that is effective in relapsing–remitting MS may be inappropriate or detrimental in progressive forms, where enhancing mitochondrial function in neurons and oligodendrocyte precursor cells is crucial. Resolving these issues necessitates the development of robust biomarkers (e.g., serum metabolomic profiles, neuroimaging) to stratify patients according to predominant metabolic pathology, monitor target engagement, and inform treatment duration and intensity. Prospective rational combination therapies, integrating conventional immunotherapies with metabolic modulators or merging glycolysis inhibitors with AMPK activators, signify the most promising avenue.

Innovations in drug delivery, such as nanoparticles and antibody–drug conjugates, can expand the therapeutic window by targeting drugs to specific pathogenic cells or the CNS. A comprehensive systems-level approach that incorporates multi-omics will uncover new metabolic hubs and forecast individual responses.

### Biomarkers and trial design for immuno-metabolic therapies

The clinical translation of immuno-metabolic therapies requires parallel development of robust biomarkers for patient stratification, target engagement monitoring, and demonstration of proof-of-mechanism, particularly in progressive MS and remyelination trials.

*Peripheral Blood Biomarkers*: Serum neurofilament light chain (sNfL) is an established biomarker of neuroaxonal damage, reflecting acute inflammation and predicting disease activity. Serum glial fibrillary acidic protein (sGFAP) is emerging as a marker of astrocytic activation more closely associated with smoldering disease and progression independent of relapse activity (PIRA). Elevated sGFAP correlates with retinal thinning and may be a superior marker of chronic neurodegeneration. Combining sNfL, sGFAP, and CSF markers (e.g., lipid-specific IgM oligoclonal bands) can differentiate inflammatory from non-active PIRA, enabling precise stratification for metabolic modulator trials.

*Metabolomics and Lipidomics*: Serum metabolomic and lipidomic profiling is an emerging tool for identifying MS biomarkers and tracking metabolic therapy effects. Longitudinal studies link decreased phosphatidylcholine levels with declines in walking speed and manual dexterity, while circulating metabolites predict sNfL and sGFAP concentrations more effectively than clinical data or gut microbiota profiles. These signatures could serve as non-invasive biomarkers to monitor disease progression and treatment response.

*Neuroimaging Biomarkers*: TSPO-PET quantifies microglial and astrocytic activation in vivo, with glial activation in normal-appearing white matter predicting cognitive decline. Dynamic [^1^⁸F]FDG PET-MRI assesses CNS glucose metabolism, with pilot data suggesting increased glycolysis in SPMS versus RRMS, potentially serving as a progression marker. Magnetic resonance spectroscopy (MRS) maps metabolic abnormalities: reduced N-acetylaspartate (NAA) reflects neuroaxonal damage, while elevated myo-inositol (mI) indicates gliosis. Accelerated increases in the mI/tNAA ratio may signal the RRMS-to-progressive transition.

*Single-Cell Approaches*: Single-cell RNA sequencing (scRNA-seq) of cerebrospinal fluid immune cells reveals distinct transcriptional states in MS, identifying expanded B cell and plasma cell compartments and pathways such as CXCL12-CXCR4 that amplify CNS inflammation. These approaches enable identification of cellular phenotypes associated with treatment response or resistance, informing patient stratification for clinical trials.

*Trial Design for Progressive MS and Remyelination*: Remyelination trials are investigating the effects of metformin and clemastine, employing functional readouts such as P100 latency changes in visual evoked potentials (VEP), as used in the ongoing CCMR trial, along with lesional magnetization transfer ratio (MTR) to assess myelin integrity. Progressive MS trials require endpoints capturing insidious disability accumulation; composite endpoints defining PIRA have demonstrated utility as sensitive measures. Event-driven designs with composite endpoints inclusive of advanced patients are being developed. Multi-arm, multi-stage designs, like those used in the STOP-MS trial (spironolactone and famciclovir) and CAR T-cell trials, are effective at testing many different treatments at once. Integrating validated biomarkers, sNfL, sGFAP, TSPO-PET, and MRS, as secondary or exploratory endpoints is critical for demonstrating target engagement and de-risking clinical implementation of immunometabolic therapies.

### Limitations of current evidence

This review has several important limitations that should be acknowledged. First, as a narrative review rather than a systematic review or meta-analysis, it lacks the formal quality assessment, exhaustive literature search, and quantitative synthesis that would permit definitive conclusions about the strength of evidence for particular metabolic targets. The selection of topics and references reflects the authors' expert judgment, and while we have sought broad thematic coverage, the exclusion of non-English articles and conference abstracts may have introduced selection bias.

Second, the immuno-metabolic framework presented here, while conceptually powerful, is supported by preclinical evidence that has not yet been consistently validated in human MS patients. Much of the mechanistic evidence derives from EAE models, which, despite their utility, incompletely recapitulate the complexity and heterogeneity of human MS, particularly the progressive phase. The metabolic profiles described for human immune cells are often inferred from small cohort studies or in vitro experiments with isolated cell populations, and the extent to which these findings reflect in vivo pathophysiology remains uncertain.

Third, the therapeutic implications discussed are largely speculative. While several agents have shown promising effects in preclinical models, the translation of these findings to clinical benefit has been inconsistent. The failure of many promising immunomodulatory strategies in progressive MS trials suggests caution in extrapolating from preclinical data. Fourth, the field of immunometabolism is rapidly evolving, and new findings may substantially alter the interpretations presented here. The metabolic plasticity of immune cells, the heterogeneity of microglial and T-cell states revealed by single-cell technologies, and the complex relationship between systemic metabolism and CNS inflammation are areas of active investigation that may lead to revision of current paradigms.

Fifth, the review does not address the potential for adverse effects of metabolic modulation, including the risk of immunosuppression, metabolic derangements, and off-target effects on non-immune cells. These safety considerations are critical for clinical translation and merit dedicated discussion that is beyond the scope of this review.

Finally, the proposed framework emphasizes metabolic reprogramming as a therapeutic strategy, but the relative contribution of metabolic dysregulation versus other pathogenic mechanisms (genetic susceptibility, EBV infection, B-cell autoimmunity, iron toxicity, mitochondrial injury) remains incompletely defined. Finally, the proposed framework underscores metabolic reprogramming as a therapeutic approach; however, it does not address the comparative impact of metabolic dysregulation. We contend that metabolism functions as a modifiable hub; however, this viewpoint should not be construed as undermining the significance of alternative therapeutic targets. We assert that metabolism serves as a modifiable hub; however, this perspective should not be interpreted as diminishing the importance of alternative therapeutic targets.

### Future research priorities

To address these challenges and limitations, future research should prioritize:

## Preclinical priorities:

1. Advancement of more representative animal models that accurately reflect the chronic, multifactorial nature of human multiple sclerosis, especially its progressive forms.
2. Systematic direct comparisons of monotherapy versus combination metabolic approaches.
3. Examination of metabolic heterogeneity among patients and across disease stages utilizing single-cell technologies.
4. Identification of metabolic vulnerabilities specific to pathogenic cells with minimal off-target effects.

## Translational priorities:

1. Validation of accessible biomarkers (serum metabolomic profiles, neuroimaging signatures) for patient stratification and treatment assessment.
2. Phase 2 proof-of-concept trials of repurposed combinations, including metformin and existing disease-modifying therapies, with metabolic-specific endpoints.
3. Development of clinical trial designs that integrate adaptive strategies to address variations in disease stages.
4. Development of innovative chemical probes and drug delivery systems to attain cellular specificity and surmount metabolic adaptability.

## Conclusion

The pharmacological strategies outlined above encompass glutaminase inhibitors to neutralize pathogenic Th17 cells, AMPK activators like metformin to promote remyelination, mTOR inhibitors to restore immune tolerance, and NAD^+^ precursors to rejuvenate mitochondrial function and present promising preclinical opportunities that require systematic clinical evaluation to determine whether they translate into meaningful benefits for MS patients.

These strategies are informed by a growing body of evidence suggesting that MS pathogenesis is inherently multifactorial, resulting from the interaction of genetic predisposition, environmental factors, B-cell autoimmunity, and localized CNS inflammation.

In this intricate environment, metabolic dysregulation functions as an amplifier and a significant modifiable target, serving as a nexus where various pathogenic signals converge and result in persistent inflammatory and neurodegenerative consequences. Transitioning from generalized immunosuppression to targeted, mechanism-based metabolic reprogramming may expand therapeutic goals beyond inflammation management to include immune regulation, neuroprotection, and repair.

By focusing on metabolism, we may potentially address both the inflammatory triggers and the bioenergetic deficiencies that sustain them; however, this hypothesis necessitates thorough clinical evaluation alongside the metabolic barriers that impede repair. This method enhances, rather than supplants, current immunomodulatory strategies, presenting the possibility for synergistic combination therapies that address MS from various perspectives concurrently. Although significant challenges exist in specificity, resistance, and biomarker development, these hurdles are manageable. Immuno-metabolic approaches aim to link disease management with improved neurological outcomes via innovative drug delivery, strategic combination approaches, and comprehensive biomarker discovery.

Despite the theoretical appeal of immuno-metabolic targeting, various factors must moderate expectations. The intricate metabolic interrelationship between immune and neural cells indicates that interventions aimed at inhibiting pathogenic cells may unintentionally compromise protective or restorative functions. Moreover, the metabolic variability noted among patients and disease stages indicates that a universal approach is unlikely to be effective. The field must therefore advance towards biomarker-driven patient stratification and personalized therapeutic regimens. The intersection of genetics, immunology, and metabolism in MS requires a systems-level approach that recognizes the disease’s complexity while utilizing metabolic pathways as significant, actionable targets for therapeutic intervention.

## Supplementary Information

Below is the link to the electronic supplementary material.

Supplementary file1 (DOCX 15 kb)

## Data Availability

No datasets were generated or analysed during the current study.

## Abbreviations

2-DG: 2-Deoxyglucose
ACC1: Acetyl-CoA Carboxylase 1
AICAR: 5-Aminoimidazole-4-carboxamide ribonucleotide
AMPK: AMP-Activated Protein Kinase
ATP: Adenosine triphosphate
BBB: Blood-brain barrier
Breg: Regulatory B Cell
CNS: Central nervous system
DHODH: Dihydroorotate dehydrogenase
DMF: Dimethyl fumarate
DMT: Disease-modifying therapy
EAE: Experimental autoimmune encephalomyelitis
ETC: Electron transport chain
FAO: Fatty acid oxidation
FAS: Fatty acid synthesis
FDA: Food and drug administration
FDG-PET: Fluorodeoxyglucose positron emission tomography
GA: Glatiramer acetate
GLS: Glutaminase
GLUT1: Glucose transporter 1
GM-CSF: Granulocyte-macrophage colony-stimulating factor
HIF-1α: Hypoxia-inducible factor 1-Alpha
IFN: Interferon
IL: Interleukin
ISG: Interferon-stimulated gene
JAK-STAT: Janus Kinase-signal transducer and activator of transcription
Keap1: Kelch-Like ECH-associated protein 1
M1-like: Classically activated (Pro-inflammatory) Macrophage/Microglia
M2-like: Alternatively activated (Anti-inflammatory/Repair) Macrophage/Microglia
MHC: Major histocompatibility complex
Th1: T Helper 1 Cell
Treg: Regulatory T Cell
MMF: Monomethyl fumarate
MS: Multiple sclerosis
mTOR: Mechanistic target of rapamycin
mTORC1: Mechanistic target of rapamycin complex 1
mTORC2: Mechanistic target of rapamycin complex 2
NAD +: Nicotinamide adenine dinucleotide
NADH: Nicotinamide adenine dinucleotide (Reduced)
NADPH: Nicotinamide adenine dinucleotide Phosphate
NF-κB: Nuclear Factor Kappa-Light-Chain-Enhancer of Activated B Cells
NLRP3: NOD-, LRR- and Pyrin Domain-Containing Protein 3
NMN: Nicotinamide mononucleotide
NO: Nitric oxide
NR: Nicotinamide riboside
Nrf2: Nuclear factor erythroid 2-related factor 2
OPC: Oligodendrocyte precursor cell
OXPHOS: Oxidative phosphorylation
PET: Positron emission tomography
PI3K: Phosphoinositide 3-Kinase
PKM2: Pyruvate Kinase M2
PML: Progressive multifocal leukoencephalopathy
PPAR: Peroxisome proliferator-activated receptor
PPMS: Primary progressive multiple sclerosis
Rho: Ras homologous
ROS: Reactive oxygen species
RRMS: Relapsing-remitting multiple sclerosis
S1P: Sphingosine-1-phosphate
SIRT1: Sirtuin 1
SPMS: Secondary progressive multiple sclerosis
TCA: Tricarboxylic acid
TGF-β: Transforming growth factor-beta
Th17: T Helper 17 Cell
VCAM-1: Vascular cell adhesion molecule 1
