# Supplementary material for: Immunometabolic reprogramming in multiple sclerosis: from pathogenic amplifier to therapeutic target in neuroinflammation and remyelination
Source: Inflammopharmacology. 2026 Jul 28;34(9):5843–72. doi: 10.1007/s10787-026-02350-y (PMC13558365; doi:10.1007/s10787-026-02350-y)
Supplement: Supplementary file 1 [file 10787_2026_2350_MOESM1_ESM.docx]

- Immunometabolic imbalance in MS: Th17 glycolysis vs. Treg FAO drives neuroinflammation and remyelination failure.
- Repurposing metabolic modulators like metformin offers a new strategy to restore immune tolerance in MS.
- Targeting glutaminolysis and AMPK/mTOR pathways could suppress chronic neuroinflammation and promote CNS repair in MS.
- Beyond immunosuppression: metabolic reprogramming of immune and neural cells emerges as a promising therapeutic axis in MS.
